# Respiratory oscillometry in individuals with fibrodysplasia ossificans progressiva

**DOI:** 10.1186/s13023-025-03846-6

**Published:** 2025-07-01

**Authors:** Anastasiia Vasileva, Joyce K. Y. Wu, Melissa Valaee, Ethan Ortiz, Zoltán Hantos, Lianne Tile, Irene Ho, Angela M. Cheung, Chung-Wai Chow

**Affiliations:** 1 Division of Respirology, Department of Medicine, University Health Network, 585 University Avenue, 11 PMB 130, Toronto, ON M5G 2N2 Canada; 2 Toronto General-Pulmonary Function Laboratory, University Health Network, Toronto, Canada; 3 Department of Anesthesiology and Intensive Therapy, Semmelweis University, Budapest, Hungary; 4 Division of General Internal Medicine, Department of Medicine, University Health Network, Toronto, Canada; 5 Osteoporosis Program, University Health Network and Sinai Health System, Toronto, Canada; 6 Temerty Faculty of Medicine, Centre of Excellence in Skeletal Health Assessment, University of Toronto, Toronto, Canada; 7 Department of Medicine, Temerty Faculty of Medicine, University of Toronto, Toronto, Canada

**Keywords:** Fibrodysplasia ossificans progressiva, Pulmonary function, Respiratory oscillometry

## Abstract

**Background:**

Fibrodysplasia ossificans progressiva (FOP) is an ultra-rare genetic bone disease that is characterized by progressive heterotopic ossification of the thoracic cavity. Prognosis is poor with cardiopulmonary complications being the main cause of death. Spirometry is a well-established metric of functional exercise capacity and prognosis in lung diseases but its use is limited in this population. Accuracy and validity of spirometry is dependent on forced expiratory maneuvers which are difficult to perform for individuals with FOP. Oscillometry is an effort-independent pulmonary function test that is highly sensitive to changes in respiratory mechanics. Little is known about oscillometry in individuals with FOP. The purpose of this paper is to characterize FOP using oscillometry.

**Results:**

Eight participants with FOP were recruited for oscillometry prior to spirometry. Cumulative Analogue Joint Involvement Scale (CAJIS) scores were used to evaluate total body and regional FOP burden. Spirometry showed a uniform pattern of restrictive physiology in all eight participants with no significant difference amongst the group. Oscillometry revealed significant diversity in respiratory mechanics and chest wall involvement from normal, airway obstruction and ventilatory inhomogeneity to extra-thoracic airflow obstruction. We compared individuals with normal and abnormal oscillometry and found no statistically significant differences in functional status and clinical parameters. However, there is a tendency for lower CAJIS scores and fewer recent flare-ups in those with more normal respiratory mechanics. The tidal volumes were significantly higher in the group with more normal respiratory mechanics. Two wheelchair-dependent participants exhibited a pattern of high respiratory resistance that increased during both inspiration and expiration, to suggest presence of a fixed extra-thoracic defect.

**Conclusions:**

Oscillometry provides additional, more detailed information beyond spirometry in individuals with FOP. It is far easier than spirometry to complete and may be useful to help track disease progression and response to therapies in individuals with FOP.

## Background

Fibrodysplasia ossificans progressiva (FOP) is a severe progressive disabling, ultra-rare genetic bone disorder that affects approximately 1/2,000,000 people world-wide [1]. Death results primarily from cardiorespiratory failure [2] due to progressive immobilization of thorax with cumulative heterotopic ossification (HO) of the chest cavity [3, 4]. With disease progression, individuals become totally dependent on diaphragmatic breathing as the diaphragm is spared [5]. Flare-ups are painful episodes of connective tissue inflammation that result in transformation of ligaments, tendons and skeletal muscles into heterotopic bone. Episodes of flare-ups start in early childhood, around age of five [6]. Progressive accumulation of HO decreases mobility, causing most individuals to become wheelchair bound by age of 30 [4, 7]. Life expectancy is 40–50 years.

Identification of the specific ALK2/ACVR1 gene variation in individuals with FOP [8] has contributed to discovery of therapeutic targets. Palovarotene, a selective retinoic acid receptor gamma agonist, has been approved for treatment of FOP since 2022. Other compounds are currently being evaluated in clinical trials with the hope that disease progression can be modified [9–12]. However, assessing the effectiveness of novel therapies is limited by the lack of easily available and reliable biomarkers to monitor clinical response and disease progression.

Pulmonary function tests (PFT) provide well established metrics of respiratory disease severity. These metrics correlate well with functional exercise capacity and are commonly used to evaluate progression and prognosis in lung diseases. Their use in individuals with FOP has been limited, as the forced expiratory maneuvers required for spirometry are difficult for this population. Limited mobility which worsens with disease progression renders moving in and out of the body box for plethysmography highly untenable. The few published studies in FOP have identified a restrictive pulmonary function defect and normal diffusing capacity for carbon monoxide (DLCO) [5, 13–15].

Oscillometry is a different modality of PFT that is performed during normal breathing. It measures the mechanical impedance of the respiratory system with external oscillations superimposed on normal quiet breathing. Oscillometry is highly sensitive to changes in respiratory mechanics [16] and has been shown to provide information that can facilitate earlier diagnosis of different lung diseases. It has been shown to detect exacerbations of asthma [17–20] and chronic obstructive lung disease [21–25] with greater sensitivity than spirometry and has been found to be highly correlated with severity of idiopathic pulmonary fibrosis [26]. Oscillometry also offers additional pragmatic advantages over spirometry, as it is completed in less than 10 min in any clinical setting where patients can breathe quietly while wearing a nose-clip.

There are no published data of oscillometry in people with FOP. The aim of this study is to characterize oscillometry and assess its relationship with FOP disease severity with regard to airway and thoracic involvement.

## Methods

This study was approved by the University Health Network (UHN) Research Ethics Board (#19–5582 and 17–5652). Participants were recruited from the UHN-FOP clinic whose catchment includes all individuals with FOP in Canada. All individuals with FOP were eligible. From July 2022 to September 2024, we enrolled 8 participants and 18 healthy volunteers for oscillometry prior to conventional PFT at Toronto General Pulmonary Function Laboratory. Spectral oscillometry using multi-frequency signals from 5 to 37 Hz was performed first, followed by 10 Hz mono-frequency oscillometry. Oscillometry was performed using the tremoflo® C-100 device (Thorasys, Canada) following published technical standards and quality control guidelines [27–31]. Spirometry was performed using the Medisoft HypAir PFS (Sorinnes, Belgium) conducted in accordance with the American Thoracic Society (ATS)/European Respiratory Society guidelines [32]. Reference values for oscillometry were derived from the Oostveen and Nowowiejska equations [33, 34] and spirometry from the Global Lung Initiative [35].

**Fig. 1:**
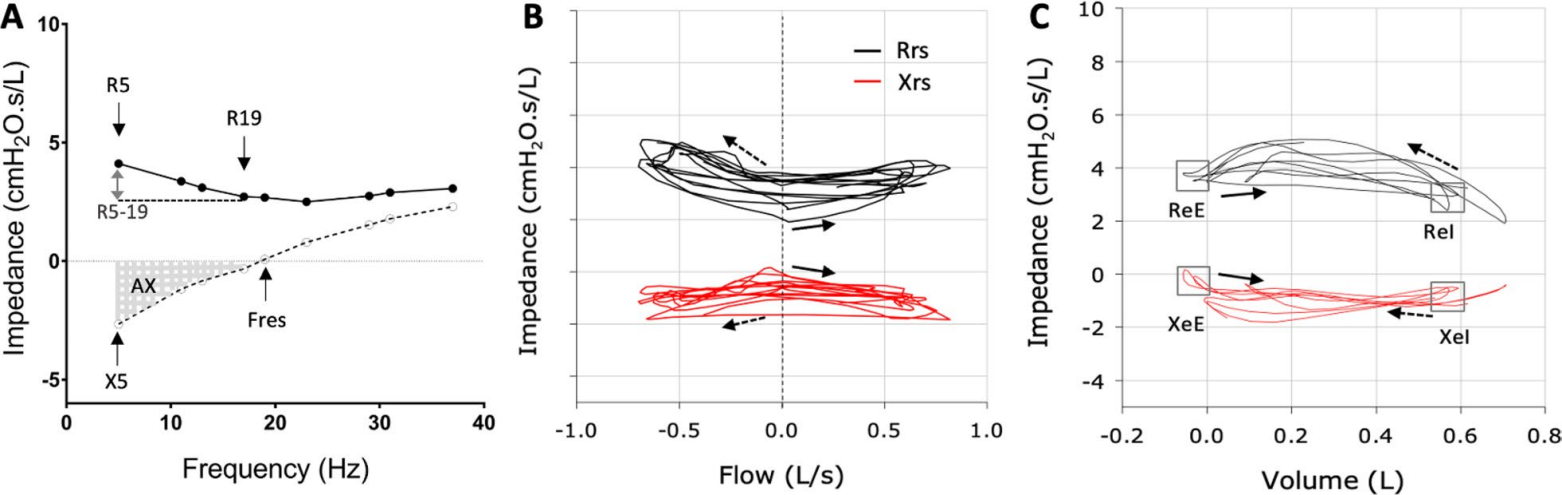
Key metrics in spectral (**A**) and mono-frequency intrabreath (**B**,** C**) oscillometry. Spectral oscillometry provides the mean values of resistance (Rrs) and reactance (Xrs) over an entire breath. The key metrics in a spectral oscillogram (**A**) are shows: R5, resistance at 5 Hz, a measure of total respiratory resistance; R5-19, difference in resistance between 5 and 19 Hz, a measure of small airway obstruction and inhomogeneity of ventilation; X5, reactance at 5 Hz which reflects the stiffness or elastance of the pulmonary and chest wall tissues; and AX, the integrative index of reactance between X5 and Fres (frequency of resonance). Intrabreath oscillometry tracks the changes in Rrs and Xrs at a single frequency (10 Hz) during breathing and is plotted against tidal volume (**B**) or flow (**C**). The resistance (R) and reactance (X) during zero flow at end expiration (eE) and end inspiration (eI) are shown. The solid arrow indicates beginning of inspiration. The dotted arrow indicated beginning of expiration

Oscillometry parameters of interest are shown (Fig. 1). Spectral oscillometry (Fig. 1A) provides the mean values of impedance at different frequencies over an entire breath and include: R5, resistance at 5 Hz, a measure of total respiratory resistance; R5-19, difference in resistance between 5 and 19 Hz, a measure of small airway obstruction and inhomogeneity of ventilation; X5, reactance at 5 Hz which reflects the stiffness or elastance of the pulmonary and chest wall tissues; and AX, the integrative index of reactance between X5 and Fres (frequency of resonance). AX is considered a more robust metric of elastance and peripheral ventilation inhomogeneity compared to X5 as it is less subject to interference from the subject’s breathing rate [28, 36]. Obstructive respiratory physiology is characterized by higher resistance at all frequencies, high R5-19, low X5 and high AX [37]. Restrictive physiology is characterized by a rightward shift of the reactance curve, leading to low X5 and high AX, while resistance is normal across the frequencies and R5-19 is low [26].

Intrabreath oscillometry tracks changes in respiratory mechanics within the breathing cycle, using a single sinusoidal signal. The key intrabreath oscillometry metrics are the measurements of resistance (R) and reactance (X) during zero flow, that is at end expiration (ReE and XeE) and end inspiration (ReI and XeI). Analysis of the intrabreath resistance and reactance loops during a tidal breath (Fig. 1B and C) provides more granular details that can help differentiate the site of airflow obstruction and parenchymal vs chest wall restriction [26, 38].

Conventional PFT parameters of interest are the forced expiratory volume in one second (FEV_1_), forced vital capacity (FVC) and FEV_1_/FVC ratio.

The cumulative analogue joint involvement scale (CAJIS) scores were used to evaluate total body and regional FOP burden [39, 40]. The CAJIS scores, ambulatory status and number of flare-up episodes within the last two years were assessed by a physician as part of routine care during the participant’s clinical visit at TGH. Imaging data (including computed tomography and plain radiographs) were reviewed and included, if available in the participant’s electronic medical records.

Exploratory statistical analysis was performed using the R Studio version 4.1.1 (R Foundation). Group comparisons were conducted using t-test for normally distributed continuous variables, Wilcoxon test for non-normal continuous variables, and Pearson’s chi-square test for categorical variables.

## Results

The mean age of the group was 26 years (range 14–39) with a mean BMI of 25.1 (range 17.7–37.4) and a total body CAJIS score (mean = 11.9, range 7–23). All participants had the classic ACVR1 R206H variation except one who had a ACVR2A variation. All participants demonstrated clinical characteristic of FOP with progressive formation of HO observed on CT images and plain radiographs. The CAJIS thoraco-lumbar spine score was similar in all participants but one who had a lower score of 1. A score of 0 means no restriction, 1 some restriction, and 2 totally ankylosed and fixed. The mean number of flare-ups in the preceding 2 years was 5.9 (range 0–12). Of the 8 participants, only 2 were fully independent, and 2 were wheelchair dependent (Table 1).

**Table 1:** Participants' characteristics at time of enrolment

ID	Sex	Age, years	BMI, kg/m^2^	CAJIS thoraco-lumbar spine score	CAJIS total body score	Flare-ups within last 2 years	Functional status	Treatment
1	M	14	37.4	2	7	6	Independent	NSAID, pain medications; prednisone for flare up—last August 2024
2	M	35	24.9	2	23	5	Can walk, wheelchair for long distance	Palovarotene; NSAID, antihistamine, pain medications; prednisone for flareup – last in May 2024
3	M	39	19.4	2	13	2	Independent	Pain medications; In randomized clinical trial; prednisone for flare up—last January 2023
4	F	21	24.0	1	7	6	Can walk with walker, wheelchair for long distance	NSAID, antihistamine, pain medications; In randomized clinical trial; prednisone for flareup – last in February 2023
5	F	29	17.7	2	17	4	Wheelchair dependent	Palovarotene until May 2024; NSAID, antihistamine, pain medications; In randomized clinical trial; prednisone for flareup – last dose April 2024
6	F	19	23.8	2	11	12	Wheelchair dependent	NSAID, antihistamine medications; In randomized clinical trial; prednisone for flareup—last June 2024
7	F	34	33.3	2	9	0	Can walk, wheelchair for long distance	NSAID, antihistamine, pain medications; In randomized clinical trial; prednisone for flareup – last in July 2024
8	F	18	20.1	2	8	12	Can walk, wheelchair for long distance	NSAID, pain medications; In randomized clinical trial; prednisone for flare up—last July 2024

Spirometry showed a pattern of restriction in all participants with proportionate reduction in FEV_1_ and FVC (mean = 56.7% and 52.1% predicted, respectively) and normal FEV_1_/FVC (mean = 92.4%; Table 2). Plethysmography was not possible due to the limited mobility from FOP and inability to get into the body box. In the overall group, oscillometry was abnormal, with the most significant abnormal being the X5 z-score and XeI when compared to healthy controls (Table 2).

**Table 2:** Respiratory oscillometry in participants with FOP and healthy controls

	FOP (n = 8)	Healthy controls (n = 18)	P-value
Age (years)	26.12 (9.30)	30.67 (10.50)	0.303
Sex, F (%)	5 (62.5)	13 (72.2)	0.972
BMI (kg/m^2^)	25.08 (6.90)	23.40 (6.31)	0.549
FEV_1_ (L)	2.26 (0.48)	3.65 (0.57)	** < 0.001**
%FEV_1_	56.71 (5.28)	92.31 (8.71)	** < 0.001**
FVC (L)	2.45 (0.56)	4.32 (0.70)	** < 0.001**
%FVC	52.12 (6.30)	95.20 (13.06)	** < 0.001**
FEV_1_/FVC	92.42 (5.62)	84.24 (2.05)	** < 0.001**
R5 *	5.08 [3.80, 6.40]	3.02 [2.73, 3.75]	**0.017**
%R5	171.76 [148.35, 205.36]	110.16 [93.52, 129.96]	**0.002**
R5 z-score	1.98 (1.26)	0.31 (0.98)	**0.001**
R19 *	4.29 [2.60, 5.10]	2.97 [2.65, 3.37]	0.267
%R19	144.31 [116.10, 161.11]	103.16 [91.35, 112.48]	**0.015**
R19 z-score	1.19 (1.26)	0.17 (0.84)	**0.021**
X5 *	− 2.44 [− 2.70, − 1.65]	− 0.95 [− 1.13, − 0.81]	**0.002**
%X5	273.18 [233.89, 311.67]	95.69 [84.61, 113.14]	**0.002**
X5 z-score	− 2.98 (1.74)	0.16 (0.99)	** < 0.001**
R5-19 *	0.79 [0.47, 1.45]	0.01 [− 0.14, 0.14]	**0.001**
AX (cmH_2_O/L)	11.11 [6.13, 15.43]	2.60 [1.93, 3.82]	**0.001**
Vt (L)	0.80 [0.65, 0.86]	0.71 [0.58, 0.85]	0.697
Fres (Hz)	16.87 [13.90, 18.69]	10.17 [9.30, 12.99]	**0.002**
ReE *	3.72 [3.20, 4.32]	2.38 [2.17, 2.84]	**0.012**
ReI *	3.32 [2.50, 3.74]	1.92 [1.72, 2.46]	**0.015**
XeE *	− 0.39 [− 0.59, − 0.07]	0.31 [0.06, 0.48]	**0.003**
XeI *	− 0.48 [− 0.72, − 0.28]	0.24 [− 0.11, 0.36]	** < 0.001**

BMI: body mass index; FEV_1_: forced expiratory volume in 1 s; FVC: forced vital capacity; X5: reactance at 5 Hz; R5: resistance at 5 Hz; R5-19: difference in resistance between 5 and 19 Hz; Fres: resonance frequency; AX: reactance area between 5 Hz and Fres; Vt: tidal volume; ReE: resistance at end-expiration; ReI: resistance at end-inspiration; XeE: reactance at end-expiration; XeI: reactance at end-inspiration. Units of measure: * cmH_2_O∙s/L. Spirometry presented as mean (SD), oscillometry as median [IQR]. Bolded *p*-values indicate statistical significance (*p* < 0.05)

**Fig. 2:**
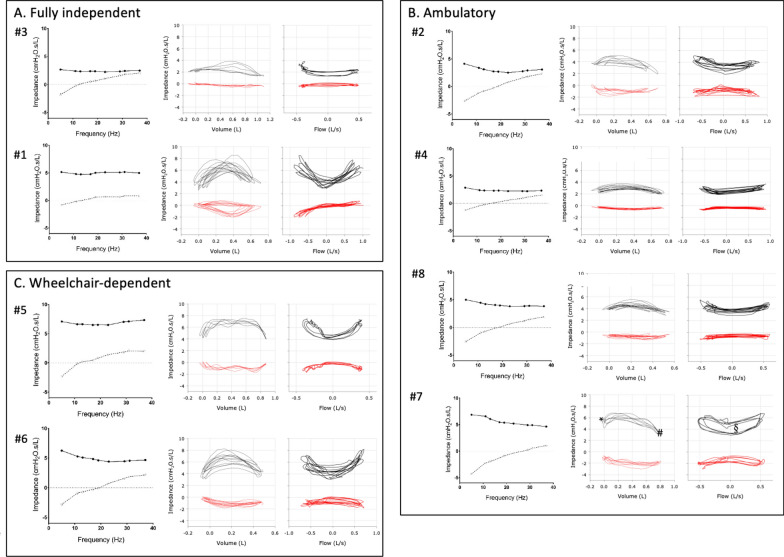
Spectral and mono-frequency intrabreath oscillometry at time of enrolment. Respiratory impedance plots from a Fully independent (**A**), Ambulatory (**B**) and Wheelchair-dependent (**C**) participants. Mean impedance data vs. frequency (left), intrabreath impedance data vs. volume (middle) and flow (right). Resistance and reactance data are plotted in solid/ black and dotted/ red, respectively.* ReE (resistance at end expiration); # ReI (resistance at end inspiration); § increased resistance vs flow loop area

However, oscillometry was highly variable amongst the 8 participants (Table 3, Fig. 2). Two participants (#1 and #4) had completely normal oscillometry with normal AX and R5-19 along with Z-scores < 1.64 for R5 and R19, and > − 1.64 for X5. While participant #1 was fully independent, participant #4 was ambulatory but needed wheelchair assistance for longer outings. The oscillometry patterns of the remaining 3 ambulatory participants (#2, #7, #8) had ventilatory inhomogeneities, as reflected by elevated R5-19 (> 1.0 cm H_2_O.s/L), low X5 (z-scores < 1.64) and high AX (> 10 cm H_2_O/L). The particularly high R5-19 and AX values in participant #7 (BMI 33.3) likely reflect the impact of obesity-related changes in respiratory mechanics as this pattern of peripheral airway obstruction has been described in obese patients [37]. Participant #3 was fully independent and had oscillometry that was remarkable for low X5 (z-score − 3.02). The two wheelchair-dependent participants (#5 and #6) had different abnormal spectral oscillometry. While both participants exhibited high resistance values (R5 z-score: 4.13 and 2.87, respectively) which are indicative of overall narrowing of the airways and increased total respiratory resistance, participant #5 had normal R5-19 and AX, reflecting homogenous ventilation. In contrast, participant #6 exhibited high R5-19 and AX which are indicative of airflow obstruction with ventilatory inhomogeneity.

**Table 3:** Spirometry and oscillometry at time of enrolment

**ID**	1	2	3	4	5^α^	6^α^	7	8
Sex	M	M	M	F	F	F	F	F
Age (years)	14	35	39	21	29	19	34	18
BMI (kg/m^2^)	37.4	24.9	19.4	24	17.7	23.8	33.3	20.1
FEV_1_ (L)	2.89	2.64	2.57	2.64	2.05	1.68	1.79	1.79
%FEV_1_	59.8	56.4	57.7	64.3	57.9	47.4	59.2	51.0
FVC (L)	3.43	2.65	2.67	2.91	2.08	1.81	1.96	2.09
%FVC	60.2	45.3	47.6	60.5	50.3	44.6	56.1	52.4
FEV_1_/FVC	84.2	99.6	96.3	90.7	98.6	93.0	91.2	85.8
R5 *	5.14	4.12	2.67	2.87	7.06	6.25	6.86	5.01
%R5	149.35	177.47	145.36	104.93	299.52	214.14	166.06	202.43
R5 z-score	0.72	2.05	1.33	0.18	4.13	2.87	1.91	2.65
R19 *	5.00	2.68	2.37	2.33	6.51	4.61	5.37	3.98
%R19	189.86	112.81	117.19	82.4	247.78	151.53	141.94	146.68
R19 z-score	1.02	0.46	0.61	− 0.78	3.64	1.67	1.4	1.54
X5 *	− 0.81	− 2.66	− 1.8	− 1.23	− 2.35	− 2.82	− 4.29	− 2.53
X5 z-score	0.33	− 4.57	− 3.02	− 1.08	− 3.48	− 3.59	− 4.65	− 3.79
%X5	81.89	354.16	282.48	157.21	306.83	263.88	259.45	326.17
R5-19 *	0.13	1.43	0.3	0.53	0.55	1.64	1.49	1.03
AX (cmH_2_0/L)	3.22	16.12	6.15	6.08	8.25	15.2	35.81	13.96
Vt (L)	0.82	0.69	0.95	0.78	0.99	0.53	0.82	0.48
Fres (Hz)	14.08	18.66	11.96	15.77	13.32	18.77	27.05	17.97
ReE *	3.51	3.45	2.08	2.44	4.54	4.25	5.44	3.94
ReI *	3.40	2.64	1.36	2.08	4.25	4.41	3.57	3.24
XeE *	0.41	− 0.55	− 0.03	− 0.29	− 0.08	− 0.50	− 1.17	− 0.73
XeI *	− 0.11	− 0.65	− 0.30	− 0.35	− 0.23	− 0.94	− 1.85	− 0.62

BMI: body mass index; FEV_1_: forced expiratory volume in 1 s; FVC: forced vital capacity; X5: reactance at 5 Hz; R5: resistance at 5 Hz; R5-19: difference in resistance between 5 and 19 Hz; Fres: resonance frequency; AX: reactance area between 5 Hz and Fres; Vt: tidal volume; ReE: resistance at end-expiration; ReI: resistance at end-inspiration; XeE: reactance at end-expiration; XeI: reactance at end-inspiration. Units of measure: * cmH_2_O∙s/L. ^α^ Patient who were wheelchair dependent

Analysis of the intrabreath oscillometry revealed normal dynamic patterns in all the ambulatory participants with minimal changes in the resistance and reactance loops during breathing (Fig. 2A and B, middle and right panels). The only exception was participant #7. The increase in ReE (denoted as *) compared ReI (denoted as #) and increased resistance vs flow loop area (denoted as §) are indicative of airflow obstruction. The two wheelchair-dependent participants and one of the fully independent individuals (#1) exhibited similar and abnormal intrabreath resistance loops showing increased resistance during both inspiration and expiration, a pattern suggestive of upper airway flow nonlinearities (Fig. 2C).

Given the different patterns of oscillometry in these 8 participants, we next explored whether those with worse respiratory mechanics had different clinical characteristics. Participants were grouped according to low AX (< 12 cmH_2_O/L, n = 4) or high AX (> 12 cmH_2_O/L, n = 4). No significant differences were observed in the clinical or spirometry patterns although there was tendency to higher CAJIS scores and more flareup in the previous 2 years in those with high AX (Table 4).

**Table 4:** Comparison of the participants with low vs high AX values

	Low AX (n = 4)	High AX (n = 4)	*P*-value
Age (years)	25.75 (10.75)	26.50 (9.26)	0.919
Sex, F (%)	2 (50)	3 (75)	1.000
BMI (kg/m^2^)	24.62 (8.92)	25.52 (5.58)	0.870
CAJIS thoraco-lumbar spine	1.75 (0.50)	2.00 (0.00)	0.356
CAJIS total body score	11.00 (4.90)	12.75 (6.95)	0.695
Flare-ups within last 2 years	4.50 (1.91)	7.25 (5.85)	0.406
Wheelchair dependent (%)	1 (25)	1 (25)	1.000
FEV_1_ (L)	2.54 (0.35)	1.98 (0.45)	0.095
%FEV_1_	59.92 (3.07)	53.50 (5.30)	0.081
FVC (L)	2.77 (0.56)	2.13 (0.37)	0.102
%FVC	54.65 (6.67)	49.60 (5.59)	0.290
FEV_1_/FVC	92.45 (6.42)	92.40 (5.69)	0.991
R5 *	4.01 [2.82, 5.63]	5.63 [4.79, 6.40]	0.564
%R5	147.35 [135.25, 186.89]	189.95 [174.62, 205.36]	0.248
R5 z-score	1.59 (1.76)	2.37 (0.46)	0.425
R19 *	3.69 [2.36, 5.39]	4.29 [3.66, 4.80]	0.773
%R19	153.53 [108.49, 204.34]	144.31 [134.66, 147.89]	0.773
R19 z-score	1.12 (1.84)	1.27 (0.55)	0.885
X5 *	− 1.51 [− 1.93, − 1.13]	− 2.74 [− 3.18, − 2.63]	**0.021**
%X5	219.85 [138.38, 288.57]	295.03 [262.78, 333.17]	0.248
X5 z-score	− 1.81 (1.77)	− 4.15 (0.54)	**0.045**
R5-19 *	0.41 [0.26, 0.54]	1.46 [1.33, 1.53]	**0.021**
AX (cmH_2_0/L)	6.12 [5.37, 6.68]	15.66 [14.89, 21.04]	**0.021**
Vt (L)	0.89 [0.82, 0.96]	0.61 [0.52, 0.72]	**0.043**
Fres (Hz)	13.70 [12.98, 14.51]	18.72 [18.49, 20.84]	**0.021**
ReE *	2.97 [2.35, 3.76]	4.09 [3.82, 4.54]	0.248
ReI *	2.74 [1.90, 3.61]	3.40 [3.09, 3.78]	0.386
XeE *	− 0.06 [− 0.13, 0.08]	− 0.64 [− 0.84, − 0.53]	**0.021**
XeI *	− 0.26 [− 0.31, − 0.20]	− 0.79 [− 1.17, − 0.64]	**0.021**

BMI: body mass index; FEV_1_: forced expiratory volume in 1 s; FVC: forced vital capacity; X5: reactance at 5 Hz; ex: during expiration; in: during inspiration; R5: resistance at 5 Hz; R5-19: difference in resistance between 5 and 19 Hz; Fres: resonance frequency; AX: reactance area between 5 Hz and Fres; Vt: tidal volume; ReE: resistance at end-expiration; ReI: resistance at end-inspiration; XeE: reactance at end-expiration; XeI: reactance at end-inspiration. Units of measure: * cmH_2_O∙s/L. Spirometry presented as mean (SD), oscillometry as median [IQR]. Bolded *p*-values indicate statistical significance (*p* < 0.05)

## Discussion

In the current study, 8 of the 20 known individuals with FOP (of whom 4 are children) living in Canada were evaluated with spirometry and oscillometry. To our knowledge, this is the first study to characterize oscillometry in FOP population. As a pulmonary function test, oscillometry offers significant advantages to the traditional tests with spirometry and plethysmography as it is conducted during normal quiet breathing and can be performed in any setting because the devices are portable. Patients prefer oscillometry over spirometry [29, 41]. Moreover, oscillometry is possible for patients who cannot perform the forced expiratory maneuvers needed for spirometry or whose mobility limits their ability to sit in the plethysmograph box.

Similar to previous studies, all participants exhibited a restrictive physiology pattern on spirometry [5, 13–15]. Surprisingly, we found diverse oscillometry patterns of respiratory mechanics in the 8 participants. They varied from normal to central airway narrowing with increased R5 to small airway obstruction with ventilatory inhomogeneity (increased R5-19 and increased AX) and extrathoracic airflow obstruction (increased resistance during both inspiration and expiration in intrabreath analysis). Intrabreath reactance measurements were found to be normal in some participants (#1, #3, and #4), suggesting that both the lung parenchyma and the chest wall tissues were unaffected. Conversely, low XeI values were observed in others (#6 and #7), which may indicate excessive stretch of the respiratory tissues. Additionally, the resistive defect observed on spirometry may also result from the chest wall. The assessment of the contribution of chest wall impedance (Zcw) to Zrs through esophageal pressure measurement was not feasible in the present investigation. Earlier studies addressing different frequency ranges [42–44] have demonstrated that, in healthy subjects, Zcw is comparable to that of the lungs.

To the best of our knowledge, no study has hitherto investigated oscillation mechanics and its components in FOP. Van Noord et al. [45] measured Zrs in patients with ankylosing spondylitis and kyphoscoliosis, and observed decreases in Xrs in both groups; however, kyphoscoliosis was also associated with a significant elevation in Rrs. Consequently, it can be inferred that, akin to the aforementioned study by Van Noord et al., the increased viscous and elastic losses observed in patients with elevated Rrs and lowered Xrs are attributable to the modified chest wall mechanics. In a related investigation, oscillometry measurements were made in a small group of patients suffering from osteogenesis imperfecta [46]. However, since the primary focus of this study was bronchial obstruction and bronchodilator response, the potential effect of altered chest wall structure on Zrs remains unclear.

Given the small sample size, meaningful comparisons could not be made about correlation of the respiratory mechanics and the CAJIS scores, particularly the thoraco-lumbar spine scores. The strong flow dependence of the intrabreath resistance in three participants suggests extrathoracic airflow obstruction. The underlying mechanism is not known. Airway and vocal cord calcification have not been reported in individuals with FOP, and were not seen in the low-dose whole body CT scans of these three participants. Calcification of oropharyngeal muscles and soft tissues or calcification impinging on the trachea or larynx could potentially lead to a fixed airway aperture but was also not seen in the CT scans of these two participants. One participant did have an unusual cord of mineralization from the hyoid anteriorly to the mandible, but it is unlikely for this cord to cause airflow obstruction. Since upper airway flow nonlinearities can be due to many causes, further studies are needed to better understand the underlying mechanism of extrathoracic airflow obstruction in affected individuals.

## Conclusions

Oscillometry is particularly advantageous in FOP as accurate measurements can still be made in people with severe functional limitations for whom the maneuvers needed for spirometry are challenging. Our data show that oscillometry provides additional information beyond spirometry regarding the respiratory status of individuals with FOP. In particular, our data reveal that participants had a range of normal to abnormal respiratory mechanics despite similar severity of restriction observed on spirometry. These findings are congruent with data in different patient populations and in several large population surveys where oscillometry was found to detect disease earlier and to be associated with burden of respiratory symptoms [17–25]. Given the ease of application, our data support the addition of oscillometry for monitoring individuals with FOP as it may be useful for tracking disease progression and response to therapies.

## Data Availability

The datasets generated during and/or analysed during the current study are available on reasonable request. Following publication of the manuscript, deidentified data will be made available on request following review, approval and completion of data sharing agreements between University Health Network and the requesting institution(s).

## References

[1] Liljesthröm M, Pignolo RJ, Kaplan FS. Epidemiology of the Global fibrodysplasia ossificans progressiva (FOP) community. J Rare Dis Res Treat. 2020;5(2):31–6.

[2] Kaplan FS, Zasloff MA, Kitterman JA, Shore EM, Hong CC, Rocke DM. Early mortality and cardiorespiratory failure in patients with fibrodysplasia ossificans progressiva. J Bone Joint Surg Am. 2010;92(3):686–91. 20194327 10.2106/JBJS.I.00705 PMC2827822

[3] Kaplan FS, Pignolo RJ, Al Mukaddam M, Shore EM. Genetic disorders of heterotopic ossification: fibrodysplasia ossificans progressiva and progressive osseous heteroplasia. In: Bilezikian J, editor. Primer on the Metabolic Bone Diseases and Disorders of Mineral Metabolism –. 9th ed. Washington, D.C.: The American Society for Bone and Mineral Research; 2019. p. 865–70.

[4] Pignolo RJ, Shore EM, Kaplan FS. Fibrodysplasia ossificans progressiva: diagnosis, management, and therapeutic horizons. Pediatric Endocrinol Rev : PER. 2013;10(Suppl 2):437–48. PMC3995352 23858627

[5] Connor JM, Evans CC, Evans DA. Cardiopulmonary function in fibrodysplasia ossificans progressiva. Thorax. 1981;36(6):419–23. 7314012 10.1136/thx.36.6.419 PMC471526

[6] Pignolo RJ, Bedford-Gay C, Liljesthrom M, et al. The natural history of flare-ups in fibrodysplasia ossificans progressiva (FOP): A comprehensive global assessment. J Bone Mineral Res : Official J Amer Soc Bone Mineral Res. 2016;31(3):650–6. 10.1002/jbmr.2728 PMC4829946 27025942

[7] Pignolo RJ, Cheung K, Kile S, et al. Self-reported baseline phenotypes from the International Fibrodysplasia Ossificans Progressiva (FOP) Association Global Registry. Bone. 2020;134: 115274. 32062004 10.1016/j.bone.2020.115274

[8] Shen Q, Little SC, Xu M, et al. The fibrodysplasia ossificans progressiva R206H ACVR1 mutation activates BMP-independent chondrogenesis and zebrafish embryo ventralization. J Clin Investig. 2009;119(11):3462–72. 19855136 10.1172/JCI37412 PMC2769180

[9] Pignolo RJ, Baujat G, Hsiao EC, Keen R, Wilson A, Packman J, Strahs AL, Grogan DR, Kaplan FS. Palovarotene for fibrodysplasia ossificans progressiva (FOP): results of a randomized, placebo-controlled, double-blind phase 2 trial. J Bone Mineral Res. 2020;37(10):1891–902. 10.1002/jbmr.4655 PMC9804935 35854638

[10] Pignolo RJ, Hsiao EC, Al Mukaddam M, Baujat G, Berglund SK, Brown MA, Brown MA, Cheung AM, De Cunto C, Delai P, Haga N, Kannu P. Reduction of new heterotopic ossification (HO) in the open-label, phase 3 MOVE trial of palovarotene for fibrodysplasia ossificans progressiva (FOP). J Bone Mineral Res. 2023;38(3):381–94. 10.1002/jbmr.4762 36583535

[11] Nikishina IP, Arsenyeva SV, Matkava VG, Arefieva AN, Kaleda MI, Smirnov AV, Blank LM, Kostik MM. Successful experience of tofacitinib treatment in patients with fibrodysplasia ossificans progressiva. Pediatr Rheumatol Online J. 2023;21(1):92. 37644581 10.1186/s12969-023-00856-1 PMC10464034

[12] Di Rocco M, Forleo-Neto E, Pignolo RJ, Keen R, Orcel P, Funck-Brentano T, Kaplan FS. Garetosmab in fibrodysplasia ossificans progressiva: A randomized, double-blind, placebo-controlled phase 2 trial. Nat Med. 2023;29(10):2615–24. 37770652 10.1038/s41591-023-02561-8 PMC10579054

[13] Kussmaul WG, Esmail AN, Sagar Y, Ross J, Gregory S, Kaplan FS. Pulmonary and cardiac function in advanced fibrodysplasia ossificans progressiva. Clin Orthop Relat Res. 1998;346:104–9. 9577416

[14] Buhain WJ, Rammohan G, Berger HW. Pulmonary function in myositis ossificans progressiva. Am Rev Respir Dis. 1974;110(3):333–7. 4415387 10.1164/arrd.1974.110.3.333

[15] Botman E, Smilde BJ, Hoebink M, et al. Deterioration of pulmonary function: An early complication in Fibrodysplasia Ossificans Progressiva. Bone Rep. 2021;14: 100758. 33748352 10.1016/j.bonr.2021.100758 PMC7972965

[16] Bates JH, Irvin CG, Farre R, Hantos Z. Oscillation mechanics of the respiratory system. Compr Physiol. 2011;1(3):1233–72. 23733641 10.1002/cphy.c100058

[17] Shi Y, Aledia AS, Tatavoosian AV, Vijayalakshmi S, Galant SP, George SC. Relating small airways to asthma control by using impulse oscillometry in children. J Allergy Clin Immunol. 2012;129(3):671–8. 22178635 10.1016/j.jaci.2011.11.002 PMC3373178

[18] Kuo CR, Lipworth B. Airwave oscillometry and patient-reported outcomes in persistent asthma. Annals Allergy Asthma Immunol. 2020;124(3):289–90. 10.1016/j.anai.2019.12.017 31904425

[19] Foy BH, Soares M, Bordas R, et al. Lung computational models and the role of the small airways in asthma. Am J Respir Crit Care Med. 2019;200(8):982–91. 31106566 10.1164/rccm.201812-2322OC PMC6794099

[20] Tang FSM, Rutting S, Farrow CE, et al. Ventilation heterogeneity and oscillometry predict asthma control improvement following step-up inhaled therapy in uncontrolled asthma. Respirology. 2020;25(8):827–35. 32026586 10.1111/resp.13772

[21] Frantz S, Nihlen U, Dencker M, Engstrom G, Lofdahl CG, Wollmer P. Impulse oscillometry may be of value in detecting early manifestations of COPD. Respir Med. 2012;106(8):1116–23. 22613172 10.1016/j.rmed.2012.04.010

[22] Aarli BB, Calverley PMA, Jensen RL, Eagan TML, Bakke PS, Hardie JA. Variability of within- breath reactance in COPD patients and its association with dyspnoea. Eur Respir J. 2015;45(3):625–34. 25359342 10.1183/09031936.00051214

[23] Dean J, Kolsum U, Hitchen P, Gupta V, Singh D. Clinical characteristics of COPD patients with tidal expiratory flow limitation. Int J Chron Obstruct Pulmon Dis. 2017;12:1503–6. 28579768 10.2147/COPD.S137865 PMC5446959

[24] Young H, Guo F, Eddy R, Maksym G, Parraga G. Oscillometry and pulmonary MRI measurements of ventilation heterogeneity in obstructive lung disease: relationship to quality of life and disease control. J Appl Physiol. 2018;125:73–85. 10.1152/japplphysiol.01031.2017. 29543132 10.1152/japplphysiol.01031.2017

[25] Eddy RL, Westcott A, Maksym GN, Parraga G, Dandurand RJ. Oscillometry and pulmonary magnetic resonance imaging in asthma and COPD. Physiol Rep. 2019;7(1): e13955. 30632309 10.14814/phy2.13955 PMC6328923

[26] Wu JK, Ma J, Nguyen L, Dehaas EL, Vasileva A, Chang E, Liang J, Huang QW, Cassano A, Binnie M, Shapera S. Correlation of respiratory oscillometry with CT image analysis in a prospective cohort of idiopathic pulmonary fibrosis. BMJ Open Respir Res. 2022;9(1): e001163. 35396320 10.1136/bmjresp-2021-001163 PMC8996008

[27] Oostveen E, MacLeod D, Lorino H, et al. The forced oscillation technique in clinical practice: methodology, recommendations and future developments. Eur Respir J. 2003;22(6):1026–41. 14680096 10.1183/09031936.03.00089403

[28] King GG, Bates J, Berger KI, Calverley P, de Melo PL, Dellacà RL, et al. Technical standards for respiratory oscillometry. Eur Respir J. 2020;55:1900753. 31772002 10.1183/13993003.00753-2019

[29] Wu JK, DeHaas E, Nadj R, Cheung AB, Dandurand RJ, Hantos Z, et al. Development of quality assurance and quality control guidelines for respiratory oscillometry in clinic studies. Respir Care. 2020;65:1687–93. 10.4187/respcare.07412. 32209708 10.4187/respcare.07412

[30] Chang E, Vasileva A, Nohra C, Ryan CM, Chow CW, Wu JKY. Conducting respiratory oscillometry in an outpatient setting. JoVE (J Visual Exper). 2022;182: e63243. 10.3791/63243 35467649

[31] Hantos, Z., Wu, J. K., Dandurand, R. J., & Chow, C. W. (2023). Quality control in respiratory oscillometry: reproducibility measures ignoring reactance?. *ERJ Open Research*, *9*(3). 10.1183/23120541.00070-2023 PMC10291313 37377657

[32] Graham BL, Steenbruggen I, Miller MR, Barjaktarevic IZ, Cooper BG, Hall GL, et al. Standardization of spirometry 2019 Update. An official American thoracic society and European respiratory society technical statement. Am J Respir Crit Care Med. 2019;200:e70-88. 10.1164/rccm.201908-1590ST. 31613151 10.1164/rccm.201908-1590ST PMC6794117

[33] Oostveen E, Boda K, van der Grinten CP, et al. Respiratory impedance in healthy subjects: baseline values and bronchodilator response. Eur Respir J. 2013;42(6):1513–23. 23598954 10.1183/09031936.00126212

[34] Nowowiejska B, Tomalak W, Radliński J, Siergiejko G, Latawiec W, Kaczmarski M. Transient reference values for impulse oscillometry for children aged 3–18 years. Pediatr Pulmonol. 2008;43(12):1193–7. 18988256 10.1002/ppul.20926

[35] Quanjer PH, Stanojevic S, Cole TJ, et al. Multi-ethnic reference values for spirometry for the 3–95-yr age range: the global lung function 2012 equations. Eur Respir J. 2012;40(6):1324–43. 10.1183/09031936.00080312. 22743675 10.1183/09031936.00080312 PMC3786581

[36] Kaminsky, D.A., Simpson, S.J., Berger, K.I., Calverley, P., De Melo, P.L., Dandurand, R., Dellacà, R.L., Farah, C.S., Farré, R., Hall, G.L. and Ioan, I., 2022. Clinical significance and applications of oscillometry. *European Respiratory Review*, 31(163). 10.1183/16000617.0208-2021 PMC9488764 35140105

[37] Bates JHT, Maksym GN. Mechanical determinants of airways hyperresponsiveness. Critical ReviewsTM Biomed Eng. 2011. 10.1615/CritRevBiomedEng.v39.i4.30. 10.1615/critrevbiomedeng.v39.i4.30 22011234

[38] Hantos Z. Intra-breath oscillometry for assessing respiratory outcomes. Curr Opin Physio. 2021;22: 100441.

[39] Kaplan FS, Al Mukaddam M, Pignolo RJ. A cumulative analogue joint involvement scale (CAJIS) for fibrodysplasia ossificans progressiva (FOP). Bone. 2017;101:123–8. 28465250 10.1016/j.bone.2017.04.015

[40] Pignolo RJ, Kaplan FS. Clinical staging of fibrodysplasia ossificans progressiva (FOP). Bone. 2018;109:111–4. 28943457 10.1016/j.bone.2017.09.014

[41] Wu JKY, Xu JJ, Vasileva A, Nohra C, Binnie M, Shapera S, Fisher JH, Ryan CM, McInnis M, Hantos Z, Chow CW. Respiratory oscillometry with CT image analysis in idiopathic pulmonary fibrosis following single lung transplant. Respir Med Case Rep. 2024;22(49): 102016. 10.1016/j.rmcr.2024.102016 PMC10973643 38559325

[42] Ferris BG Jr, Mead J, Opie LH. Partitioning of respiratory flow resistance in man. J Appl Physiol. 1964;19(4):653–8. 14195575 10.1152/jappl.1964.19.4.653

[43] Nagels J, Landser FJ, Van der Linden L, Clement J, Van de Woestijne KP. Mechanical properties of lungs and chest wall during spontaneous breathing. J Appl Physiol. 1980;49(3):408–16. 7204163 10.1152/jappl.1980.49.3.408

[44] Hantos Z, Daroczy B, Suki B, Galgoczy G, Csendes T. Forced oscillatory impedance of the respiratory system at low frequencies. J Appl Physiol. 1986;60(1):123–32. 2935519 10.1152/jappl.1986.60.1.123

[45] Van Noord JA, Cauberghs M, Van de Woestijne KP, Demedts M. Total respiratory resistance and reactance in ankylosing spondylitis and kyphoscoliosis. Eur Respir J. 1991;4(8):945–51. 1783085

[46] Storoni S, Verdonk SJ, Micha D, Jak PM, Bugiani M, Eekhoff EM, van den Aardweg JG. Bronchial obstruction in osteogenesis imperfecta can be detected by forced oscillation technique. Front Med. 2023;10:1301873. 10.3389/fmed.2023.1301873 PMC10764585 38179272

